# Co-occurrence of colistin-resistance genes *mcr-1* and *mcr-3* among multidrug-resistant *Escherichia coli* isolated from cattle, Spain, September 2015

**DOI:** 10.2807/1560-7917.ES.2017.22.31.30586

**Published:** 2017-08-03

**Authors:** Marta Hernández, M Rocío Iglesias, David Rodríguez-Lázaro, Alejandro Gallardo, Narciso M Quijada, Pedro Miguela-Villoldo, Maria Jorge Campos, Segundo Píriz, Gema López-Orozco, Cristina de Frutos, José Luis Sáez, María Ugarte-Ruiz, Lucas Domínguez, Alberto Quesada

**Affiliations:** 1 Laboratorio de Biología Molecular y Microbiología, Instituto Tecnológico Agrario de Castilla y León, Valladolid, Spain; 2 Departamento de Ingeniería Agrícola y Forestal, Tecnología de los Alimentos, Escuela Técnica Superior de Ingenierías Agrarias, Universidad de Valladolid, Palencia, Spain; 3 These authors contributed equally to the manuscript; 4 Departamento de Bioquímica, Biología Molecular y Genética, Facultad de Veterinaria, Universidad de Extremadura, Cáceres, Spain; 5 Área de Microbiología, Departamento de Biotecnología y Ciencia de los Alimentos, Universidad de Burgos, Burgos, Spain; 6 VISAVET Health Surveillance Centre, Universidad Complutense, Madrid, Spain; 7 MARE - Marine and Environmental Sciences Centre, Instituto Politécnico de Leiria, Peniche, Portugal; 8 Departamento de Sanidad Animal, Facultad de Veterinaria, Universidad de Extremadura, Cáceres, Spain; 9 Subdirección General de Sanidad e Higiene Animal y Trazabilidad, Ministerio de Agricultura y Pesca, Alimentación y Medio Ambiente, Madrid, Spain; 10 Laboratorio Central de Veterinaria, Ministerio de Agricultura y Pesca, Alimentación y Medio Ambiente, Algete, Spain; 11 Departamento de Sanidad Animal, Facultad de Veterinaria, Universidad Complutense, Madrid, Spain.; 12 INBIO G+C, Universidad de Extremadura, Cáceres, Spain

**Keywords:** mcr-3, mcr-1, colistin, antimicrobial resistance, cattle, bovine, Escherichia coli

## Abstract

Colistin resistance genes *mcr-3* and *mcr-1* have been detected in an *Escherichia coli* isolate from cattle faeces in a Spanish slaughterhouse in 2015. The sequences of both genes hybridised to same plasmid band of ca 250 kb, although colistin resistance was non-mobilisable. The isolate was producing extended-spectrum beta-lactamases and belonged to serotype O9:H10 and sequence type ST533. Here we report an *mcr*-3 gene detected in Europe following earlier reports from Asia and the United States.

Very recently, in June 2017, Yin et al. detected a third mobile colistin resistance gene *mcr-3* on an IncHI2-type plasmid, pWJ1, in a porcine *E. coli* isolate from Malaysia [1]. The authors also identified similar elements in a shotgun genome sequence of a human *Klebsiella pneumoniae* isolate from Thailand and a human *Salmonella enterica* serovar Typhimurium isolate from the United States [1].

We found an *Escherichia coli* isolate carrying the *mcr-3* gene among other isolates expressing colistin resistance. It was sampled in cattle faeces at the time of slaughter in Spain in September 2015. The aim of this paper is to describe the presence of *mc*r-3 in Europe in a strain also carrying the *mcr*-1 gene. 

## Screening of bovine samples for colistin-resistant bacteria 

The VISAVET Health Surveillance Centre in Madrid has been carrying out the national surveillance for detection of extended-spectrum beta-lactamase (ESBL)-producing bacteria in food-producing animals since 2014, commissioned by the Spanish Ministry of Agriculture and Fishing, Food and Environment according to Commission Implementing Decision 2013/652/EU [2]. The screening was performed at slaughterhouses during 2015 on healthy cattle younger than one year from 318 farms (caecal content of 636 animals). The procedure followed the EURL-AR recommendations for detecting ESBL-producing *E. coli* [3]. 

A total of 152 samples (47.8%) were suspected to be positive, so antimicrobial susceptibility testing was performed by Sensititre microbroth dilution using EUVSEC and EUVSEC2 plates (Trek Diagnostic Systems, US) to confirm their beta-lactamase production. The antimicrobial drugs to be included in each panel are detailed in the Commission Implementing Decision 2013/652/EU [2]. Six *E. coli* isolates were found resistant to colistin and further characterised. Among them, five were PCR-positive for *mcr-*1 [4] and one isolate (ZTA15/01169–1EB1) was also PCR-positive for *mcr*-3 [1]. All isolates presented multi-resistant phenotypes (Table 1) and lacked the *mcr*-2 gene [5].

**Table 1:** Colistin resistance genes and antimicrobial resistance of *Escherichia coli* isolates of bovine origin, Spain, September 2015 (n = 6)

	ZTA15/ 01169–1EB1	ZTA15/ 00213–1EB1	ZTA15/ 00579–1EB1	ZTA15/ 01425–1EB1	ZTA15/ 01928–1EB1	ZTA15/ 02163–1EB1
Presence of *mcr* gene						
*mcr-1*	Yes	No	Yes	Yes	Yes	Yes
*mcr-3*	Yes	No	No	No	No	No
Antimicrobial resistance (minimal inhibitory concentrations)						
COL	4 (R)	4 (R)	4 (R)	4 (R)	4 (R)	4 (R)
CIP	> 8 (R)	8 (R)	> 8 (R)	> 8 (R)	8 (R)	8 (R)
NAL	> 128 (R)	> 128 (R)	> 128 (R)	> 128 (R)	> 128 (R)	> 128 (R)
AMP	> 64 (R)	> 64 (R)	> 64 (R)	> 64 (R)	> 64 (R)	> 64 (R)
FEP	> 32 (R)	4 (R)	> 32 (R)	16 (R)	> 32 (R)	16 (R)
FOT	> 4 (R)	> 4 (R)	> 4 (R)	> 4 (R)	> 4 (R)	> 4 (R)
FOT2	> 64 (R)	16 (R)	> 64 (R)	64 (R)	> 64 (R)	> 64 (R)
FOX	4	16 (R)	8	8	4	4
TAZ	> 8 (R)	> 8 (R)	8 (R)	4 (R)	8 (R)	8 (R)
TAZ2	8 (R)	128 (R)	16 (R)	4 (R)	8 (R)	8 (R)
TRM	16	16	8	8	8	≤ 4
ETP	0.03	≤ 0.015	0.03	0.03	0.06	0.03
IMI	≤ 0.12	≤ 0.12	≤ 0.12	0.25	0.25	0.25
MER	≤ 0.03	≤ 0.03	≤ 0.03	≤ 0.03	≤ 0.03	≤ 0.03
MER2	≤ 0.03	≤ 0.03	≤ 0.03	≤ 0.03	≤ 0.03	≤ 0.03
AZI	64 (R)	64 (R)	8	4	≤ 2	≤ 2
CHL	> 128 (R)	128 (R)	128 (R)	32 (R)	8	128 (R)
GEN	> 32 (R)	≤ 0.5	≤ 0.5	≤ 0.5	≤ 0.5	≤ 0.5
TET	> 64 (R)	64 (R)	32 (R)	> 64 (R)	64 (R)	> 64 (R)
SMX	> 1,024 (R)	> 1,024 (R)	> 1,024 (R)	> 1,024 (R)	> 1,024 (R)	> 1,024 (R)
TMP	> 32 (R)	0.5	> 32 (R)	> 32 (R)	> 32 (R)	> 32 (R)
TGC	≤ 0.25	≤ 0.25	≤ 0.25	≤ 0.25	≤ 0.25	≤ 0.25

AMP: ampicillin; AZI: azithromycin; CHL: chloramphenicol; CIP: ciprofloxacin; COL: Colistin; ETP: ertapenem; FEP: Cefepime; FOT/FOT2: cefotaxime; FOX: cefoxitin; GEN: gentamicin; IMI: imipenem; MER/MER2: meropenem; NAL: nalidixic acid; SMX: sulfamethoxazole; TAZ/TAZ2: ceftazidime; TET: tetracycline; TGC: tigecyclin; TMP: trimethoprim; TRM: temocillin.

Antibiotics are ordered according to importance/clinical impact in food-producing animals.

Resistance is indicated by (R).

*mcr-1* and mcr-3 genes were detected by PCR using previously described primers and conditions [1,4]. Minimal inhibitory concentrations were determined by using the two-fold broth microdilution reference method according to ISO 20776–1:2006 [6]. The interpretation of the quantitative data was performed as described by the Commission Implementing Decision 2013/652/EU [2], EURL-AR (EU Reference Laboratory for antimicrobial resistance in the context of animal health and food safety) and EUCAST (The European Committee on Antimicrobial Susceptibility Testing (EUCAST) [7].

Both *mcr-1* and *mcr-3* genes were detected by PCR using previously described primers and conditions [1,4]. Minimal inhibitory concentrations were determined by using the two-fold broth microdilution reference method according to ISO 20776–1:2006 [6]. The interpretation of the quantitative data was performed as described by the Commission Implementing Decision 2013/652/EU [2], EURL-AR (the EU Reference Laboratory for antimicrobial resistance in the context of animal health and food safety) and The European Committee on Antimicrobial Susceptibility Testing (EUCAST) [7]. 

The isolate ZTA15/01169–1EB1 carrying both *mcr-1* and *mcr-3* was resistant to most antimicrobial drugs analysed, including ampicillin, azithromycin, cefepime, cefotaxime, ceftazidime, chloramphenicol, ciprofloxacin, colistin, gentamicin, nalidixic acid, sulfamethoxazole, tetracycline and trimethoprim. The isolate was sensitive to carbapenems, cefoxitin, temocillin and tigecyclin (Table 1).

## Characterisation of the *mcr-*1 and *mcr-*3 *Escherichia coli* isolate 

DNA from isolate ZTA15/01169–1EB1 was extracted with the QIAGEN DNeasy Blood and Tissue Kit and sequencing libraries were prepared using the Nextera XT kit and sequenced on a MiSeq (Illumina) using v3 reagents with 2 x 300 cycles. This isolate produced 547,226 reads that were assembled using SPAdes v 3.9.0 [8]. The draft genome of 5,115,727 bp was composed by 495 contigs (N50 = 23,843, 29X coverage) and genome annotation was performed by using Prokka [9]. The profiles of serotype O9:H10, ST533, rST 30316, cgST 47043 and wgST 49795 were predicted by using EnteroBase (http://enterobase.warwick.ac.uk). The resistome of the draft genome was analysed by blastn [10] searches against the ResFinder database [11]. The presence of putative plasmids was evaluated by blastn searches against the PlasmidFinder database, revealing 100% identity to sequence probes from IncHI2 and IncI1 replicons (Table 2) [12]. Both colistin resistance genes carried by isolate ZTA15/01169–1EB1, *mcr-1* and *mcr-3*, are plasmidic and have been associated with HincHI2 plasmids [1,13].

**Table 2:** Resistome and plasmid profiles of *Escherichia coli* ZTA15/01169–1EB1, Spain, September 2015

Sequences	Coverage^a^	Identity (%)	AN^b^
*aac(3)-Iid*	1–861/861	99.884	EU022314
*aadA1*	1–972/972	97.428	X02340
*aadA2*	1–792/792	99.747	JQ364967
*blaCTX-M-55*	1–876/876	100	GQ456159
*blaTEM-1A*	1–854/861	100	HM749966
*dfrA1*	1–474/474	100	JQ690541
*floR*	1–1214/1215	98.188	AF118107
*mcr-1*	1–1626/1626	100	KP347127
*mcr-3*	1–1626/1626	99.94	KY924928
*mph(A)_1*	1–906/906	100	D16251
*mph(A)_2*	1–921/921	99.675	U36578
*strA*	1–804/804	100	M96392
*strB*	1–837/837	100	M96392
*sul1*	1–927/927	100	CP002151
*sul3*	1–792/792	100	AJ459418
*tet(A)*	1–1200/1200	100	AJ517790
IncHI2 (*repHI2*)	1–327/327	100	BX664015
IncI1_1_Alpha (RNAI-I1)	1–142/142	100	AP005147

Resistance and plasmid determinants were identified against the ResFinder and PlasmidFinder databases, respectively [9,12].

^a^ Number of query nucleotides found in the obtained draft genome compared to the total length of each reference gene sequence deposited in the ResFinder and/or PlasmidFinder databases.

^b^ GenBank accession number.

*mcr-1* was found in a 2,074 bp-length contig, and blastn comparison against the National Center for Biotechnology Information (NCBI) database [14] revealed best match with the IncHI2-type plasmid pECJS-59–244 previously described [13]. *mcr-3* was found in a 4,098 bp-length contig, and blastn of the gene showed 100% coverage (1–1626/1626) and 99,94% nucleotide identity to *mcr*-3. A unique polymorphism (C1463T) was found in its coding sequence, giving rise to a T488I variant of the protein encoded by this gene allele, hereafter named *mcr-*3.2. Moreover, isolate ZTA15/01169–1EB1 contained the mutations S83L and D87N of *Gyr*A, in complete concordance with the phenotypic results (Table 1), in addition to two beta-lactamase-encoding genes (blaCTX-M-55 and blaTEM-1) and several other resistance determinants (Table 2). CTX-M is a widely spread ESBL that could be encoded by IncHI2 and HincI1, among other plasmids [13].

Plasmid location of the *mcr-1* and *mcr-*3.2 genes from isolate ZTA15/01169–1EB1 was evidenced by nuclease S1 digestion and pulsed-field gel electrophoresis (PFGE), followed by transfer of DNA to nylon membranes and hybridisation to Dig-labelled probes (Sigma, US). Specific signals obtained by using probes for both *mcr-1* and *mcr-3*, matched a plasmid band of ca 250 kb (Figure). Specificity of the *mcr*-1 probe was evidenced by using a previously characterised strain carrying *mcr-1* on a 30 kb IncX4 plasmid [15]. A second ca 75 kb plasmid was identified by PFGE in isolate ZTA15/01169–1EB1 (Figure). However, despite the plasmidic location of the *mcr-1* and *mcr-*3.2 genes, colistin resistance was not mobilisable by conjugation in standard conditions (overnight mating at 37 °C) to the receptor strain *E. coli* J53 after selection in medium with sodium azide (100 mg/L) and colistin (2 mg/L). The previously described *E. coli* isolate ZTA14/01057 was used as a positive control in parallel, and conjugation to the same recipient was successful with 4.2·10^−2^ efficiency [16].

**Figure:**
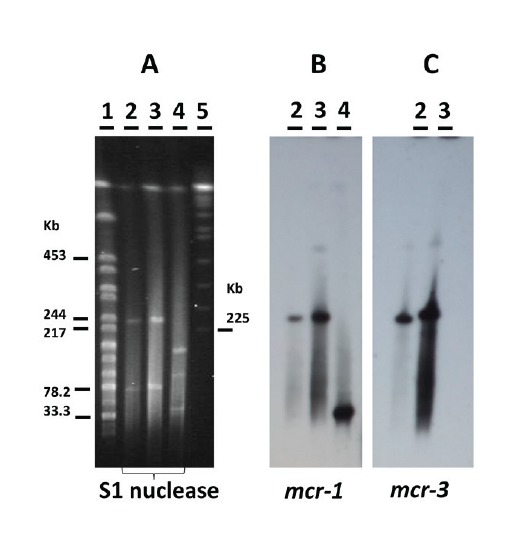
S1 nuclease mapping of *mcr-1* and *mcr-3.2* genes in *Escherichia coli* ZTA15/01169–1EB1, Spain, September 2015

A. PFGE was performed in a Bio-Rad CHEF-DRII electrophoresis system, and agarose plugs were prepared according to manufacturer instructions. *Xba*I and S1 nuclease treatments were performed as previously described [17]. Lane 1: *Xba*I-digested *Salmonella* Braenderup; Lanes 2 and 3: S1 nuclease-digested *E. coli* ZTA15/01169 at 1× (10^8^ cells/mL plug) and 3× concentration, respectively; Lane 4: S1 nuclease-digested *E. coli* HSP38 (carrying *mcr-1* by IncX4 plasmid) [15]; Lane 5: *Saccharomyces cerevisiae* chromosomes as a second molecular weight marker (Bio-Rad, US).

B. Southern hybridisation to Dig-labelled probes (Sigma, US) from *mcr-1* sequences.

C. Southern hybridisation to Dig-labelled probes (Sigma, US) from *mcr-3* sequences.

## Discussion and conclusions

The first plasmid-mediated polymyxin resistance mechanism, *mcr-1*, was reported in 2016 by Liu et al. in human *E. coli* and *K. pneumoniae* collected from five provinces in China between April 2011, and November 2014 [4]. A second resistance gene, *mcr-2*, was identified in porcine and bovine *E. coli* in Belgium in June 2016 [5], and as recently as in June 2017, Yin et al. reported the finding of the third gene, *mcr-3*, in a porcine *E. coli* isolate from Malaysia and two humans isolates of *K. pneumoniae* and *S. enterica* serovar Typhimurium from Thailand and the United States, respectively [1]. We also demonstrated in 2016 the presence of *mcr-1* in *E. coli* and *S. enterica* isolates from poultry and swine in Spain [15]. In addition to these findings, this work describes the results of screening for multidrug-resistant *E. coli* (including polymyxin resistance) of bovine origin in Spain. Among the colistin-resistant isolates found, three genotypes were identified: strains carrying *mcr*-1 alone, strains carrying *mcr*-1 and *mcr-*3.2, and strains without any plasmidic determinants. This study shows the appearance of the colistin-resistant *mcr*-3 gene in Europe as early as in 2015, as well as the coexistence of two plasmid-mediated colistin resistance genes, *mcr*-1 and *mcr-*3.2 in the same cells of isolate ZTA15/01169–1EB1.

Whole genome sequencing of isolate ZTA15/01169–1EB1 revealed *mcr-1* upstream a complete PAP2 gene in a 2,074 bp contig that showed 99.96% coverage and 100% identity to the IncHI2-type plasmid pECJS-59–244 (243,572 bp; 10). The *mcr-*3.2 gene was positioned in a 4,098 bp contig, sharing 100% coverage and 99.94% identity with the *mcr-3* gene located in the IncHI2-type plasmid pWJ1 (261,119 nt) previously described [1].

Most *mcr-1*, *mcr-2* and *mcr-3* genes are plasmidic sequences [1,4,5]. Although the PFGE and further hybridisation with *mcr-1-* and *mcr-3*-specific probes did not exclude the possibility of independent carriage by two different similar-sized plasmids, genome sequencing of isolate ZTA15/01169–1EB1 only identified the IncHI2 replicon as an appropriate candidate to harbour colistin resistance genes. We therefore assume that both genes were located on the same plasmid in our isolate.

Further efforts are focused on investigating the structure of plasmids, transmission potential, gene expression and stability of the *mcr-1* and *mcr-*3.2 genes. Furthermore, the reason why co-occurrence of *mcr*-1 and *mcr*-3 genes confers low colistin resistance needs to be elucidated.

## References

[1] Yin W Li H Shen Y Liu Z Wang S Shen Z Novel plasmid-mediated colistin resistance gene mcr-3 in Escherichia coli. MBio. 2017;8(3):e00543-17. 10.1128/mBio.00543-17 28655818 PMC5487729

[2] European Commission. Commission implementing decision 2013/652/EU on the monitoring and reporting of antimicrobial resistance in zoonotic and commensal bacteria. Official Journal of the European Union. Luxembourg: Publications Office of the European Union. 14.11.2013:L 303. Available from: http://eur-lex.europa.eu/legal-content/EN/TXT/PDF/?uri=OJ:L:2013:303:FULL&from=EN

[3] Hasman H, Agersø Y, Hendriksen R, Cavaco LM, Guerra-Roman B. Isolation of ESBL-, AmpC- and carbapenemase-producing E. coli from caecal samples. Laboratory Protocol. Version 4. Lyngby: DTU Foof; Jan 2017. Available from: http://www.eurl-ar.eu/data/images/protocols/esbl_ampc_cpeprotocol_version_caecal_january2017_version4.pdf

[4] Liu YY Wang Y Walsh TR Yi LX Zhang R Spencer J Emergence of plasmid-mediated colistin resistance mechanism MCR-1 in animals and human beings in China: a microbiological and molecular biological study. Lancet Infect Dis. 2016;16(2):161-8. 10.1016/S1473-3099(15)00424-7 26603172

[5] Xavier BB Lammens C Ruhal R Kumar-Singh S Butaye P Goossens H Identification of a novel plasmid-mediated colistin-resistance gene, mcr-2, in Escherichia coli, Belgium, June 2016. Euro Surveill. 2016;21(27):30280. 10.2807/1560-7917.ES.2016.21.27.30280 27416987

[6] International Organization for Standardization (ISO). Clinical laboratory testing and in vitro diagnostic test systems -- Susceptibility testing of infectious agents and evaluation of performance of antimicrobial susceptibility test devices -- Part 1: Reference method for testing the in vitro activity of antimicrobial agents against rapidly growing aerobic bacteria involved in infectious diseases. ISO 20776-1:2006. Geneva: ISO; 2006. Available from: https://www.iso.org/standard/41630.html

[7] European Committee on Antimicrobial Susceptibility Testing (EUCAST). Antimicrobial wild type distributions of microorganisms. Växjö: EUCAST. [Accessed: July 2017]. Available from:https://mic.eucast.org/Eucast2/SearchController/search.jsp?action=init

[8] Bankevich A Nurk S Antipov D Gurevich AA Dvorkin M Kulikov AS SPAdes: a new genome assembly algorithm and its applications to single-cell sequencing. J Comput Biol. 2012;19(5):455-77. 10.1089/cmb.2012.0021 22506599 PMC3342519

[9] Seemann T Prokka: rapid prokaryotic genome annotation. Bioinformatics. 2014;30(14):2068-9. 10.1093/bioinformatics/btu153 24642063

[10] Zhang Z Schwartz S Wagner L Miller W A greedy algorithm for aligning DNA sequences. J Comput Biol. 2000;7(1-2):203-14. 10.1089/10665270050081478 10890397

[11] Zankari E Hasman H Cosentino S Vestergaard M Rasmussen S Lund O Identification of acquired antimicrobial resistance genes. J Antimicrob Chemother. 2012;67(11):2640-4. 10.1093/jac/dks261 22782487 PMC3468078

[12] Carattoli A Zankari E García-Fernández A Voldby Larsen M Lund O Villa L In silico detection and typing of plasmids using PlasmidFinder and plasmid multilocus sequence typing. Antimicrob Agents Chemother. 2014;58(7):3895-903. 10.1128/AAC.02412-14 24777092 PMC4068535

[13] Li R Xie M Zhang J Yang Z Liu L Liu X Genetic characterization of mcr-1-bearing plasmids to depict molecular mechanisms underlying dissemination of the colistin resistance determinant. J Antimicrob Chemother. 2017;72(2):393-401. 10.1093/jac/dkw411 28073961

[14] NCBI Resource Coordinators Database Resources of the National Center for Biotechnology Information. Nucleic Acids Res. 2017;45(D1):D12-7. 10.1093/nar/gkw1071 27899561 PMC5210554

[15] Sánchez-Benito R, Iglesias MR, Quijada NM, Campos MJ, Ugarte-Ruiz M, Hernández M, et al. Escherichia coli ST167 carrying plasmid mobilisable mcr-1 and blaCTX-M-15 resistance determinants isolated from a human respiratory infection. Int J Antimicrob Agents. 2017;S0924-8579(17)30181-4. (Forthcoming). 28599866 10.1016/j.ijantimicag.2017.05.005

[16] Quesada A Ugarte-Ruiz M Iglesias MR Porrero MC Martínez R Florez-Cuadrado D Detection of plasmid mediated colistin resistance (MCR-1) in Escherichia coli and Salmonella enterica isolated from poultry and swine in Spain. Res Vet Sci. 2016;105:134-5. 10.1016/j.rvsc.2016.02.003 27033921

[17] Nonaka L Maruyama F Onishi Y Kobayashi T Ogura Y Hayashi T Various pAQU plasmids possibly contribute to disseminate tetracycline resistance gene tet(M) among marine bacterial community. Front Microbiol. 2014;5(5):152. 24860553 10.3389/fmicb.2014.00152 PMC4026752

